# Supplementary figures and images for: Effects of iNOS in Hepatic Warm Ischaemia and Reperfusion Models in Mice and Rats: A Systematic Review and Meta-Analysis
Source: Int J Mol Sci. 2022 Oct 7;23(19):11916. doi: 10.3390/ijms231911916 (PMC9569681; doi:10.3390/ijms231911916)

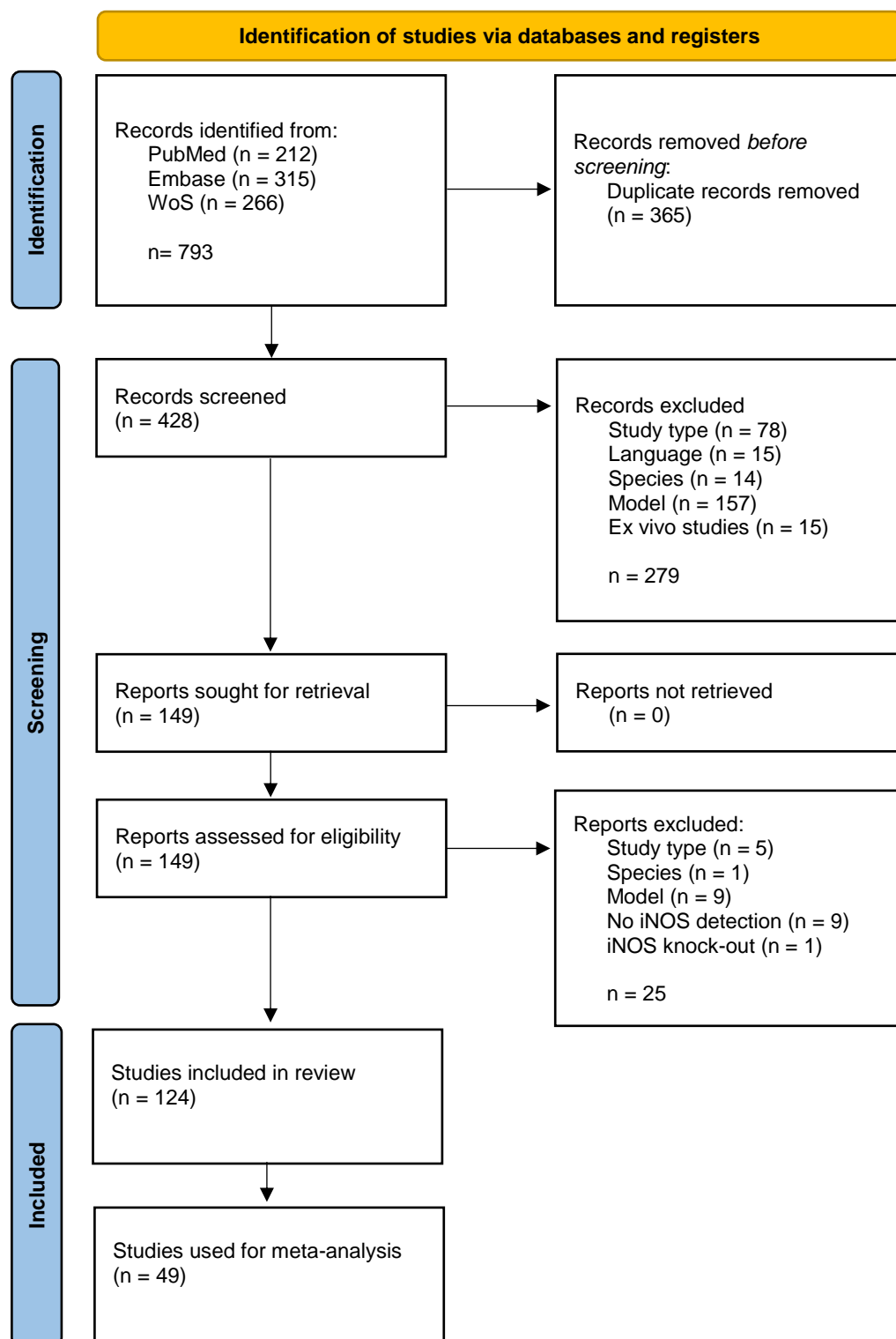

Supplement: Supplementary file 1 [file ijms-23-11916-s001.zip › ijms-1935786-supplementary/PRISMA_2020_flow_diagram_new_SRs_v1.pdf]
